# Therapeutic Potential of Vasoactive Intestinal Peptide and its Derivative Stearyl-Norleucine-VIP in Inflammation-Induced Osteolysis

**DOI:** 10.3389/fphar.2021.638128

**Published:** 2021-05-05

**Authors:** Michal Eger, Tamar Liron, Sahar Hiram-Bab, Zamzam Awida, Eliezer Giladi, David Dangoor, Mati Fridkin, David Kohavi, Illana Gozes, Yankel Gabet

**Affiliations:** ^1^ Department of Anatomy and Anthropology, Sackler Faculty of Medicine, Tel Aviv University, Tel Aviv, Israel; ^2^ Department of Prosthodontics, Goldschleger School of Dental Medicine, Sackler Faculty of Medicine, Tel Aviv University, Tel Aviv, Israel; ^3^ Department of Cell and Developmental Biology, Sackler Faculty of Medicine, Tel Aviv University, Tel Aviv, Israel; ^4^ Department of Human Molecular Genetics and Biochemistry, Elton Laboratory of Molecular Neuroendocrinology, Sackler Faculty of Medicine, Adams Super Center for Brain Studies, Tel Aviv University, Tel Aviv, Israel; ^5^ Department of Organic Chemistry, The Weizmann Institute of Science, Rehovot, Israel; ^6^ Sagol School of Neuroscience, Tel Aviv University, Tel Aviv, Israel

**Keywords:** periodontitis, periimplantitis, aseptic implant loosening, implant wear debris, topical agents, implant infection, osteoclasts

## Abstract

The common use of dental and orthopedic implants calls for special attention to the immune response leading to peri-prosthetic bone loss and implant failure. In addition to the well-established microbial etiology for oral implant failure, wear debris and in particular titanium (Ti) particles (TiP) in the implant vicinity are an important trigger of inflammation and activation of bone resorption around oral and orthopedic implants, presenting an unmet medical need. Here, we employed bacterial-derived lipopolysaccharides (LPS) to model infection and TiP to model aseptic inflammation and osteolysis. We assessed inflammation *in vitro* by measuring *IL1β*, *IL6* and *TNFα* mRNA expression in primary macrophages, osteoclastogenesis in RANKL-induced bone marrow derived pre-osteoclasts and osteolysis *in vivo* in a mouse calvarial model. We also assessed the trans-epithelial penetrability and safety of the tested compound in rats. Our results show that a lipophilic super-active derivative of vasoactive intestinal peptide (VIP), namely stearyl-norleucine-VIP (SNV) presented superior anti-inflammatory and anti-osteoclastogenic effects compared to VIP *in vitro*. In the bacterial infection model (LPS), SNV significantly reduced IL1β expression, while VIP increased IL6 expression. In the aseptic models of osteolysis, SNV showed greater suppression of *in vitro* osteoclastogenesis than VIP, and significantly inhibited inflammation-induced osteolysis *in vivo*. We also observed that expression levels of the VIP receptor VPAC-2, but not that of VPAC-1, dramatically decreased during osteoclast differentiation. Importantly, SNV previously shown to have an increased stability compared to VIP, showed here significant trans-epithelial penetration and a clean toxicological profile, presenting a novel drug candidate that could be applied topically to counter both aseptic and infection-related bone destruction.

## Introduction

The common use of dental and orthopedic implants calls for special attention to the immune response leading to peri-prosthetic bone loss and implant failure. In dentistry, peri-implantitis is a recent but already major clinical concern and the main cause of long-term implant failure (Esposito et al., 2005; Heitz-Mayfield et al., 2014; Heitz-Mayfield and Mombelli, 2014). Triggered by specific oral bacteria, it consists of an inflammatory process that leads to bone resorption (osteolysis) around dental implants (Mombelli et al., 2012). Once the process starts it can hardly be controlled and often results in implant loss (Esposito et al., 2012). Treatments usually begin with attempts to preserve the implant by mechanical cleaning of the surrounding oral flora using ultrasonic-scaling and local and systemic antibiotic administration (Heitz-Mayfield et al., 2014; Heitz-Mayfield and Mombelli, 2014). When these options fail to restrain the destructive process, the implant is surgically removed.

In addition to the well-established microbial etiology for oral peri-implant and periodontal bone loss (peri-implantitis and periodontitis, respectively), wear debris and in particular titanium (Ti) particles (TiP) in the implant vicinity are an important trigger of inflammation and activation of bone resorption. Whereas Ti is a biocompatible material, which does not induce an immune response, previous reports suggested that ions and particles shedding from implant alloys may do the opposite (Revell, 2008; Eger et al., 2017). Particles of Ti (TiP), among other metals released from the implant surface, cause an inflammatory response strikingly similar to that induced by lipopolysaccharides (a model for bacterial infection) in macrophages (Eger et al., 2018). These pro-inflammatory cytokines include IL1β, IL6 and TNFα, which are known to have a key role in stimulating bone resorption around teeth and implants (Onodera et al., 1993; Weingart et al., 1994; Vallés et al., 2008a; Vallés et al., 2008b).

Aseptic loosening due to particle debris is among the main causing factors for hip joint implant failure (Revell, 2008). These particles accumulate in the environment of the implant and induce a strong inflammatory response that eventually leads to bone resorption. High concentrations of TiP were also found around failed implants (Lee et al., 2012). With time, the gradual increase in the number of particles reaches a critical concentration that leads to periprosthetic osteolysis and may jeopardize implant survival (Goodman et al., 2014).

Vasoactive intestinal peptide (VIP) is a 28 amino-acid hydrophilic peptide (Gozes, 2008) that may inhibit both inflammation (Gozes, 2008) and osteoclastogenesis (Mukohyama et al., 2000), thus portraying this peptide as a promising candidate in the management on inflammation-induced osteolysis. It acts as a neurohormone and neurotransmitter and is involved in multiple physiological actions such as vasodilation, bronchodilation, cell division, and neuroprotection (Gozes, 2008).

VIP derivatives and conjugates were designed to include a lipophilic moiety or a shortened VIP chain (Gozes and Fridkin, 1992; Gozes et al., 1994; Gozes et al., 1995; Gozes et al., 1996; Gozes et al., 1999). Stearyl-norleucine-VIP (Stearyl-Nle^17^VIP, SNV) is a lipophilic compound consisting of a VIP molecule with an N-terminal attachment of stearic acid to increase cell and tissue bioavailability and an exchange of the oxidation-vulnerable methionine at position 17 with the stable norleucine. As cited above, SNV is 1000-fold more potent than VIP in terms of neuroprotection and cGMP formation (Ashur-Fabian et al., 1999) with a 15 min compared to ∼30 s half-life (Gozes et al., 1994; Gozes et al., 1996), and hence tested here for its therapeutic potential in inflammation-induced osteolysis. Furthermore, the stearyl lipophilic moiety allows for better skin penetration as previously described (Gozes et al., 1994), and as shown in detail below.

## Materials and Methods

All procedures involving animals were carried out in accordance with the guidelines of Tel Aviv University and were approved by the Institutional Animal Care and Use Committee (permit number M-015–047).

Cell culture. Primary bone marrow-derived macrophages (BMDMs) were isolated from the femora and tibiae of 10-week-old C57BL/6J mice (Envigo, Israel), as previously described (Hiram-Bab et al., 2015). Briefly, cells were cultured overnight at 37°C in a humidified atmosphere with 5% CO_2_ in our “standard medium” consisting of alpha-modified Eagle’s medium (αMEM, Life Science Technology, NY, United States) and 10% fetal bovine serum (FBS, Rhenium, Ltd., Modi’in, Israel). After 24 h, the non-adherent fraction was cultured in 10-cm non-culture-treated dishes containing standard medium and 100 ng/ml macrophage colony stimulating factor (M-CSF) (Takeshita et al., 2000). The resulting adherent BMDMs were collected after 3 days for the specific assays described below.

Particle generation. To obtain Ti particles that correspond to the particles shedding from oral implants during routine scaling, we subjected Ti discs that were made from Ti6Al4V (AlphaBio Tec., Petah-Tikva, Israel) to ultrasonic (US) scaling (Newtron Led, Satelec, Acteon, Marignac, France), adjusted to a frequency of 32 kHz. Particles were obtained from discs with a sand-blasted and acid-etched (SLA) surface topography as described previously (Eger et al., 2017). All particles were generated in a sterile environment. Each disc was subjected to US scaling for 60 s in distilled water (ddH_2_O), then cleaned twice with ethanol, and finally resuspended in distilled water. We previously showed that each 6 mm diameter disc generates ∼2.54 million particles on average. In all our *in vitro* assays and for the preparation of the fibrinogen-thrombin membranes (see below) we used a particle density of 1293 particles/mm^2^.

MTT assay. For proliferation assay, macrophages were plated (4000 cells/well) into 96-well plate with standard medium supplemented with 20 ng of M-CSF. Cells were treated as indicated with either TiP, SNV, VIP (10^−6^ M) or vehicle at first day of incubation. Cells viability was determined using dimethylthiazol-diphenyl-tetrazolium-bromide (MTT) after 1, 3 or 5 days. MTT was added to a final concentration of 5 mg/ml and incubated for 4 h at 37°C. After complete solubilization of the die in DMSO, plates were read at 570 nm in a colorimetric plate reader.

RNA isolation, and RT-qPCR. Following a 24 h incubation with Ti particles (or LPS/vehicle only/SNV/VIP in addition to Ti particles), macrophages were washed with sterile PBS, and RNA was extracted using Tri-RNA Reagent (Favorgen Biotech Corp, Kaohsiung, Taiwan). The 260/280 absorbance ratio was measured to verify the RNA purity and concentration. cDNA was produced using a high-capacity cDNA reverse transcription kit (Invitrogen, Grand Island, NY, United States), and real-time PCR was performed using Kapa SYBR Fast qPCR (Kapa Biosystems, Wilmington, MA, United States) on a StepOne real-time PCR machine (Applied Biosystems, Grand Island, NY, United States).

The following primers were used: F-GAAATGCCACCTTTTGACAGTG and R-TGGATGCTCTCATCAGGACAG for mouse IL1β; F-TAGTCCTTCCTACCCCAATTTCC and R-TTGGTCCTTAGCCACTCCTTC for mouse IL6; F-TCTTCTCATTCCTGCTTGTGG and R-GGTCTGGGCCATAGAACTGA for mouse TNFα; F-CCGTAACTGCACTGAAGA-AG and R-CTGTTGCTGCTCATCCATAC for VPAC-1; F-CAGCAGACCAGGAAACAT-AA and R-GCCACACGCATCTATGAA for VPAC-2 and F-ACCCAGAAGACTGTGGATGG and R-CACATTGGGGGTAGGAACAC for Gapdh. The reaction was subjected to 40 cycles of amplification using the following program: 95°C for 20 s, 60°C for 20 s, and 72°C for 25 s. The relative mRNA expression levels of the selected genes were normalized to the level of Gapdh.

Peptide synthesis. Stearyl-Nle^17^-VIP (SNV). Stearyl–HSDAVFTDNYTRLRKQ-Nle-AVKKYLNSILN-NH2, a derivative of VIP was synthesized as before (Gozes et al., 1994; Gozes et al., 1996).

Osteoclastogenesis assay. Preosteoclasts, prepared like the BMDMs, were plated in 96-well plates (7,000 cells per well, for TRAP staining, see below) or in 6-well plates (200,000 cells per well, for RNA) in standard medium supplemented with 20 ng/ml M-CSF and 50 ng/ml RANKL (R&D Systems, Minneapolis, MN, United States). After 48 h, the medium was replaced by the conditioned medium of BMDM, supplemented with RANKL and M-CSF. Where indicated, 10^−6^ M SNV or VIP were added. After 30 h, cells were stained using a TRAP kit (Sigma-Aldrich, St. Louis, MO, United States), and multinucleated (>3 nuclei) TRAP-positive cells were defined as osteoclasts. Images were acquired at an original magnification of ×4 (Evos FLC, Life Technologies, MS, United States). The number of osteoclasts and the total osteoclast area were measured using ImageJ software (National Institutes of Health, Bethesda, MD, United States).

Toxicology. We performed toxicology studies for SNV that consisted of acute subcutaneous, intravenous and oral toxicity in rats, acute dermal toxicity in rabbits, skin sensitization using adjuvant and patch test in guinea pigs, and single and repeated (for 13 weeks) dose toxicity studies in rats. The latter was a 90-days repeated dose toxicity study, consisting of SNV once a day at three different doses to the penis and vagina of male and female rats, respectively. The compound was tested for the treatment of erectile dysfunction. The active dose (7 µg SNV) was chosen as the lowest dose for the study. The highest dosage group in the repeated dose toxicity study received 3,500 µg (Gozes et al., 1994). For study 001 (parts 1 through 3), the 1× dose consisted of 0.5 mg SNV dissolved in 7.1 ml vehicle (3.55 ml 10% Sefsol [glycerin monocaprylate] + 3.55 ml isopropanol) and 100µl/animal were administered; the 1000X dose consisted of 91 mg SNV dissolved in 1.3 ml vehicle (0.65 ml 10% Sefsol +0.65 ml isopropanol) and 100µl/animal were administered. For studies 002 through 004, the indicated doses of SNV (Table 1) were dissolved in 0.25 ml 10% Sefsol +0.25 ml 40% isopropanol (vehicle). The methodology for each of the toxicology studies is detailed in Table 1.

**TABLE 1:** Toxicology studies.

Study	Model	Treatment groups	Reactions	Mortality	Conclusion
001–1 acute subcutaneous toxicity in rats	Rats (Sprague Sawley, S.D., Levinstein, Yokneam, Israel). *N* = 6 males (225–275 g) *N* = 6 females (150–210 g) in each group. Injection close to penile of vaginal tissue. Study duration: 7 acclimation days, 14 observation days	1. Saline	Necrosis of abdominal (male only) or penile skin on day 13–14 after injection. Diarrhea in 2 males of group 4 (1000X) 5–24 after injection, lasting for 2 days. The same group showed significant weight loss until 8th day, returning to original weight on day 12–14.	None	Under the conditions of this study, the acute median lethal subcutaneous dose of SNV was found to be greater the 7 mg/rat, which is the maximal practical dose
		2. Vehicle*			
		3. SNV – active dose- 1 × 7 µg/rat*			
		4. SNV (1000X) 7 mg/rat*			
		*Preparation + vehicle (100 µl/animal)			
		1X dose: 0.5 mg SNV +3.55 ml 10% sefsol (glycerin monocaprylate) + 3.55 ml isopropanol			
		1000X dose: 91 mg SNV +0.65 ml 10% sefsol +0.65 ml isopropanol			
001–2 acute intravenous toxicity in rats	As in study 001–1, except injection was intravenous (tail vein)	As in study 001–1, except injection was intravenous	Necrotic reaction was observed at the site of injection in 55% group 2, 58% group 3 and 100% group 4. Most surviving rats displayed normal body weight, and some weight loss observed initially was recovered during the study	7 rats (3 males and 4 females) out of 12 died within 3 h after administration in group 4 (SNV -1000X). One rat died in group 2 within 5–24 h after administration. No mortality occurred in groups 1 and 3	Under the conditions of this study, the acute intravenous median lethal dose of SNV was estimated to be 7 mg/male rat, and due to the higher mortality in females, it was estimated to be less than 7 mg for males and females combined. In view of necrotic reactions in group 2 – vehicle, isopropanol was reduced from 50 to 20% in the other studies (002–004)
	*N* = 6 males (160–350 g)				
	*N* = 6 females (155–285 g), for each of the experimental groups				
001–3 acute oral toxicity in rats	As in study 001–1	As in study 001–1 only test substances were administered orally using a metal catheter	None	None	Under the conditions of this study, the acute median oral lethal dose of SNV was found to be greater the 7 mg/rat, which is the maximal practical dose
	*N* = 6 males (195–275 g)				
	*N* = 6 females (155–290 g), in each experimental group				
002 acute dermal toxicity in rabbits	*N* = 8 albino female rabbits, 2.38–2.75 kg, (Weizmann Institute of Science). After shaving the fur on the back, a sterile gauze was applied and test material injected into the gauze	1. Saline (0.5 ml/site) (*N* = 2)	The 1000X dose of SNV caused slight barely perceptible erythema in 50% of the rabbits, which disappeared within 72 h following application	None	Under the conditions of this study, single dermal application of the vehicle did not cause irritation. The 1000X dose transient effect was barely perceptible
		2. Vehicle** (*N* = 2)			
		3. SNV (1000X, *N* = 4) 7 mg/site**			
		** test material + vehicle was prepared as follows (studies 002–004			
		0.25 ml 10% sefsol +0.25 ml 40% isopropanol)			
003 skin sensitization: Adjuvant and patch test	Guinea pigs (Hartley, Levinstein, Yokneam, Israel)	1. Vehicle**	The positive control group developed severe erythema, edema and necrosis, which covered most of the shaved area and beyond it. In contrast, the vehicle and test substance groups showed only slight erythema and edema within the application site	None	The study was designed to assess the degree of skin sensitization resulting from intradermal Freund’s complete adjuvant and patch application of SNV. The study showed that both SNV and vehicle alone have no skin sensitization properties
		2. SNV – (1000X) 7 mg/animal**			
	*N* = 3 males (325–352 g)	3. Positive control – 1%-chloro-2,4-dinitrobenzene in dibutyl-phthalate			
	*N* = 3 females (250–310 g)/per each of the 3 test groups	Daily applications for 3 days			
004–1 repeated dose toxicity in rats: a 13-weeks study	The repeated daily dose toxicity of SNV administered topically on the sex organs was investigated in 80 specific pathogen free (SPF) S.D. rats divided in 4 groups of 10 males and 10 females (Harlan Olac Ltd., United Kingdom)	1. SNV – active dose: 1 × 7 µg/rat**	No related adverse effects were detected throughout the study. Clinical signs were: penile edema and erythema, yellow staining of the penis, bleeding from the preputium or vagina, abscessation in the abdominal area close to the sex organ. Most of these signs were transient. One male developed transient diarrhea, which disappeared after a week. Penile edema, erythema and staining were seen in the treatment groups, but the incidence and severity of the clinical signs were not dose-related and are more probably related to the repeated handling of the rats	None. Only one death took place in a male from the low dose group due to massive abdominal hemorrhage caused by a nephroblastoma	No dose related or sex related biologically meaningful treatment effects were detected for any of the hematology or clinical chemistry parameters tested. Under the conditions of this study, daily topical application of SNV for 13 weeks did not cause any serious adverse effects at any of the doses tested
		2. SNV – 0.7 mg/rat**			
		3. SNV – 3.5 mg/rat**			
		4. Vehicle only**			
005 assessment of mutagenic potential in histidine auxotrophs of *Salmonella typhimurium* (Ames test)	Study location: Life Science Research Israel ltd. Ness Ziona	The tested SNV concentrations were 0.3–312.5 µg/standard bacterial plate	No significant increases in revertant colony numbers over control with test material at doses ranging from 0.3 to 312.5 µg/plate		Under the conditions of this study, the test material, SNV was devoid of mutagenic activity

Although, the studies were not carried out under Good Laboratory Practices (GLP) stipulations, SNV was synthesized under GLP conditions, and all the work was carried out at Tel Aviv University under meticulous conditions using professional staff and established methods (in a designated air-conditioned, clean room and specific luminary flow hood), which all strengthen the trustworthiness of the study results. Ames tests were carried out by Life Science Research Israel Ltd. Ness Ziona, Israel.

Rat model of transepithelial penetrability. Because of the high hydrophobicity of SNV, we employed an iodination labeling protocol based on the Chloramine T method (Markwell, 1982) using 1 mCi Na^125^I with a few modifications that included 1) the dissolution of SNV in dimethylformamide (DMF), 2) replacement of the phosphate buffer with 0.2 M HEPES (pH = 7.6), a more compatible buffer, and 3) reaction termination by the addition of sodium metabisulfite and KI. The pharmacokinetic profile of ^125^I-SNV absorption and distribution was evaluated following vaginal delivery of the compound to rats. Wistar rats at the estrus phase received 50 µl (7.0 µCi/rat, 15 million CPM, 100 Ci/mM specific activity) of ^125^I-SNV dissolved in 5% Sefsol, applied directly in the vagina, using a P100 micro pipettor. At the indicated time points, we anesthetized the rats with chloral hydrate and collected blood before cervical dislocation. We then harvested the uterus, liver, lungs, heart, intestine, kidneys and vagina (duplicate samples were taken from each organ). Organs were weighed and radioactivity levels were measured using a gamma counter. Each point on the chart represents at least two rats.

**FIGURE 1:**
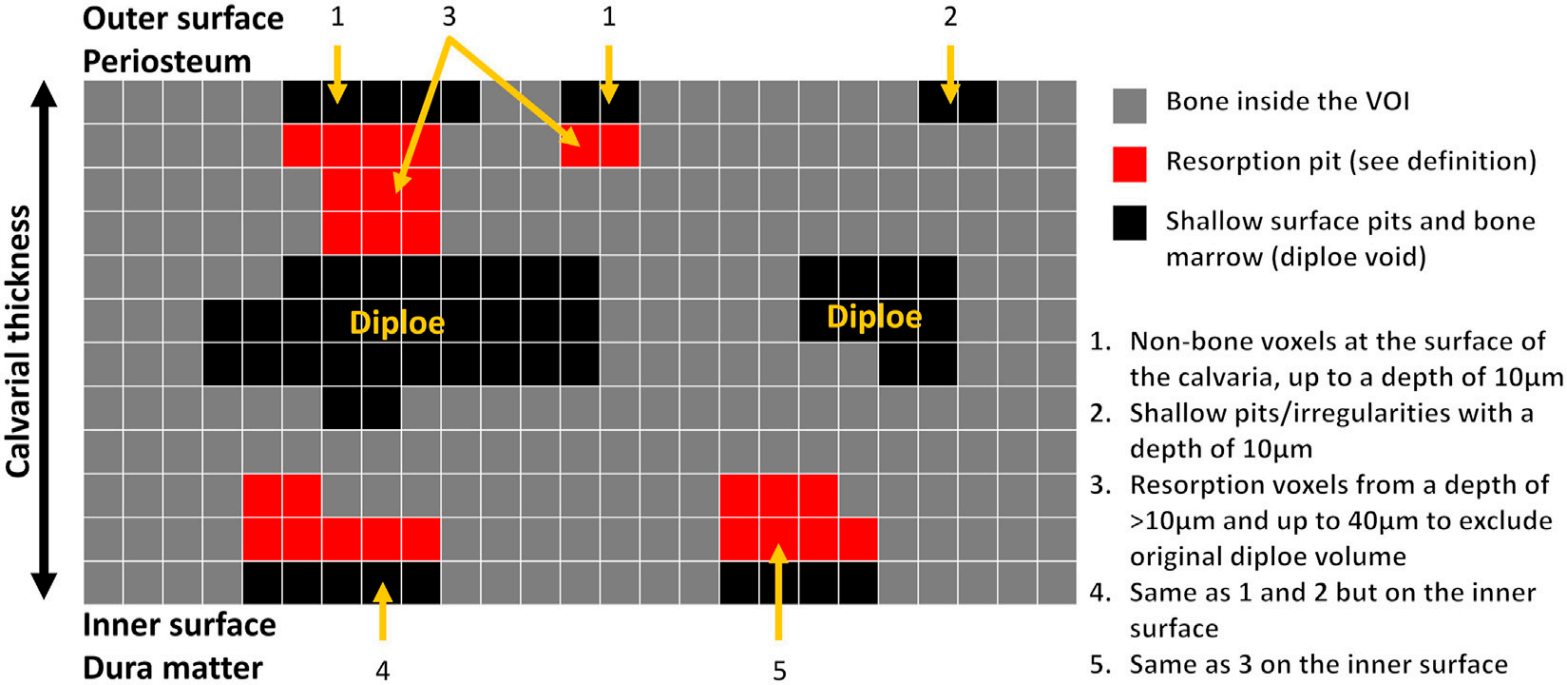
Schematic representation of image processing and quantitation of the pit resorption volume in the mouse calvaria using µCT. Note that the calvarial thickness measurement in the volume of interest (VOI) is independent of the bone marrow and pit resorption volume.

Animal model and micro-computed tomography (μCT). We used our calvarial model, as described previously (Eger et al., 2017). Briefly, US-released TiP (from SLA-treated discs) were incorporated into a fibrinogen-thrombin degradable membrane used as a scaffold to localize the TiP, and membranes with no particles were prepared as positive and negative controls, respectively. As indicated, 2 × 10^–8^ mol SNV (or saline as control) were incorporated into the membrane together with the TiP. The parietal bones of the 10-week-old C57Bl/6J female mice were exposed, and the periosteum was removed before inserting the fibrinogen-thrombin membranes to cover both parietal bones. In the control group an empty fibrinogen-thrombin membrane was inserted (with no TiP). All groups comprised six animals. Animals were euthanized 5 weeks post insertion, and the skull of each mouse was removed, fixed for 24 h in 4% phosphate-buffered formalin, followed by 70% ethanol. All specimens were scanned and analyzed using a μCT system (μCT 50, Scanco Medical AG, Switzerland). Scans were performed at a 10-μm resolution in all three spatial dimensions, with 90 kV energy, 88 μA intensity, and 1000 projections at a 1000 msec integration time. The volume of interest (VOI) was defined as two 3.7 mm circles in the center of the parietal bones. A custom-made algorithm, based on Image-Processing Language (IPL, Scanco Medical), was developed to isolate the resorption pits, defined as unmineralized volumes that were 10–40 μm deep on the bone surface (as in (Eger et al., 2017), Figure 1). Morphometric parameters were determined at the 3D level inside a fixed VOI total volume (TV) and included the total volume of the bone resorption pits (Pit Resorption Volume, PRV, mm^3^), and the calculated PRV/TV ratio (Figure 1). We also measured the mean calvarial plate thickness using a mask that obliterated the bone marrow volume and resorption pits (Figure 1). This parameter is therefore independent of the resorption volume and it discriminates between actual osteolysis (no change in the calvarial plate thickness) and irregular bone thickening (increase in the calvarial plate thickness). The values used for each animal are the average of the right and left sides.

Statistical analyses. Values are expressed as the mean ± SD unless otherwise indicated. Statistical analyses were performed using GraphPad Prism 7.0 (La Jolla, CA, United States). As all presented data typically display a normal distribution, *t*-test (between two groups) or analysis of variance (ANOVA) with Tukey’s post hoc test (multiple group comparison) were used. Differences between groups were defined as significant at *p* < 0.05.

## Results

### SNV Presents Anti-inflammatory Effects in Cell Cultures

**FIGURE 2:**
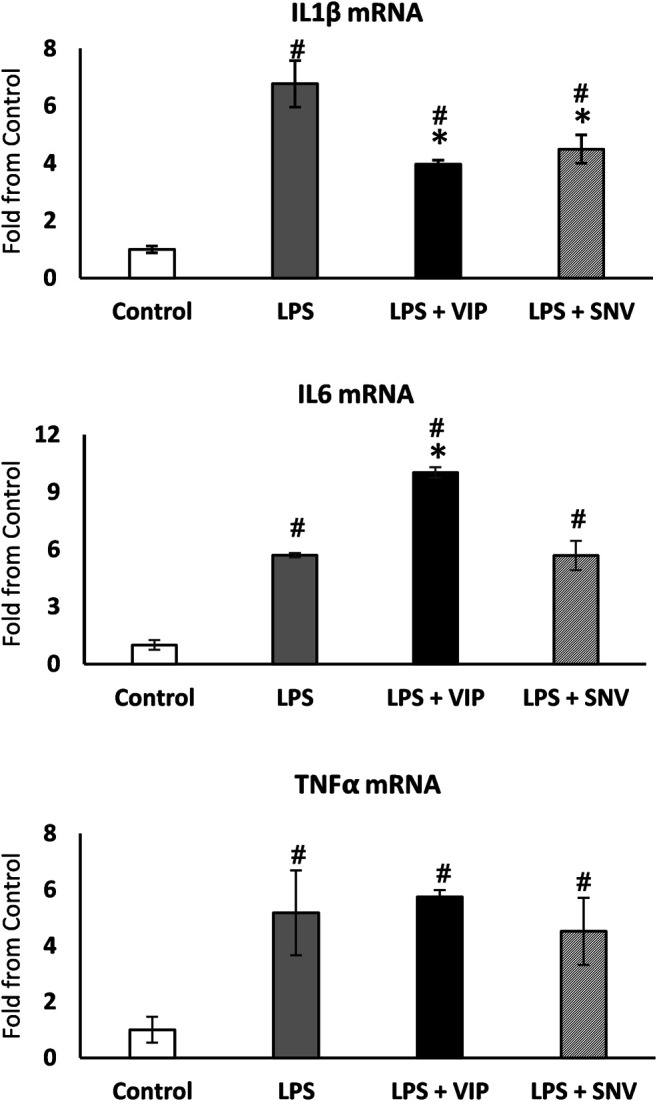
VIP and SNV displayed a partial anti-inflammatory effect in LPS-stimulated macrophage cultures. Primary murine macrophages were treated with 10^-6^M of VIP or SNV (or saline) and exposed to 1 µg/ml LPS for 24 h. Expression of IL1β, IL6 and TNFα was examined using RT-qPCR and presented as fold from untreated controls (no LPS). *n*=5, # *p*<0.05 vs. Control; *p<0.05 vs. LPS, 1-way ANOVA.

IL1β, IL6 and TNFα are the main mediators of Ti particle-mediated inflammation and osteolysis (Eger et al., 2017; Eger et al., 2018). The immuno-modulating role of VIP is well established (Harmar et al., 2012) and we first examined the effect of both VIP and SNV on the expression of these pro-inflammatory cytokines in macrophages. We treated bone marrow-derived macrophages with 10^−6^ M VIP or SNV (or saline control) 1 h before adding LPS (1 μg/ml). Control cultures were left untreated (M-CSF only). After 24 h, we extracted RNA and observed a significant increase in the expression levels of the three cytokines in the LPS-treated vs. control macrophage cultures. VIP demonstrated mixed immunomodulatory effects, decreasing IL1β but increasing IL6 (–40% and +90%, respectively, *p* < 0.05, Figure 2) and presenting no change on TNFα expression. SNV had no effect on IL6 and TNFα expression levels but it significantly reduced the expression levels of IL1β (by 35%, *p* < 0.05, Figure 2), suggesting a net anti-inflammatory effect.

### SNV Suppresses Osteoclastogenesis

**FIGURE 3:**
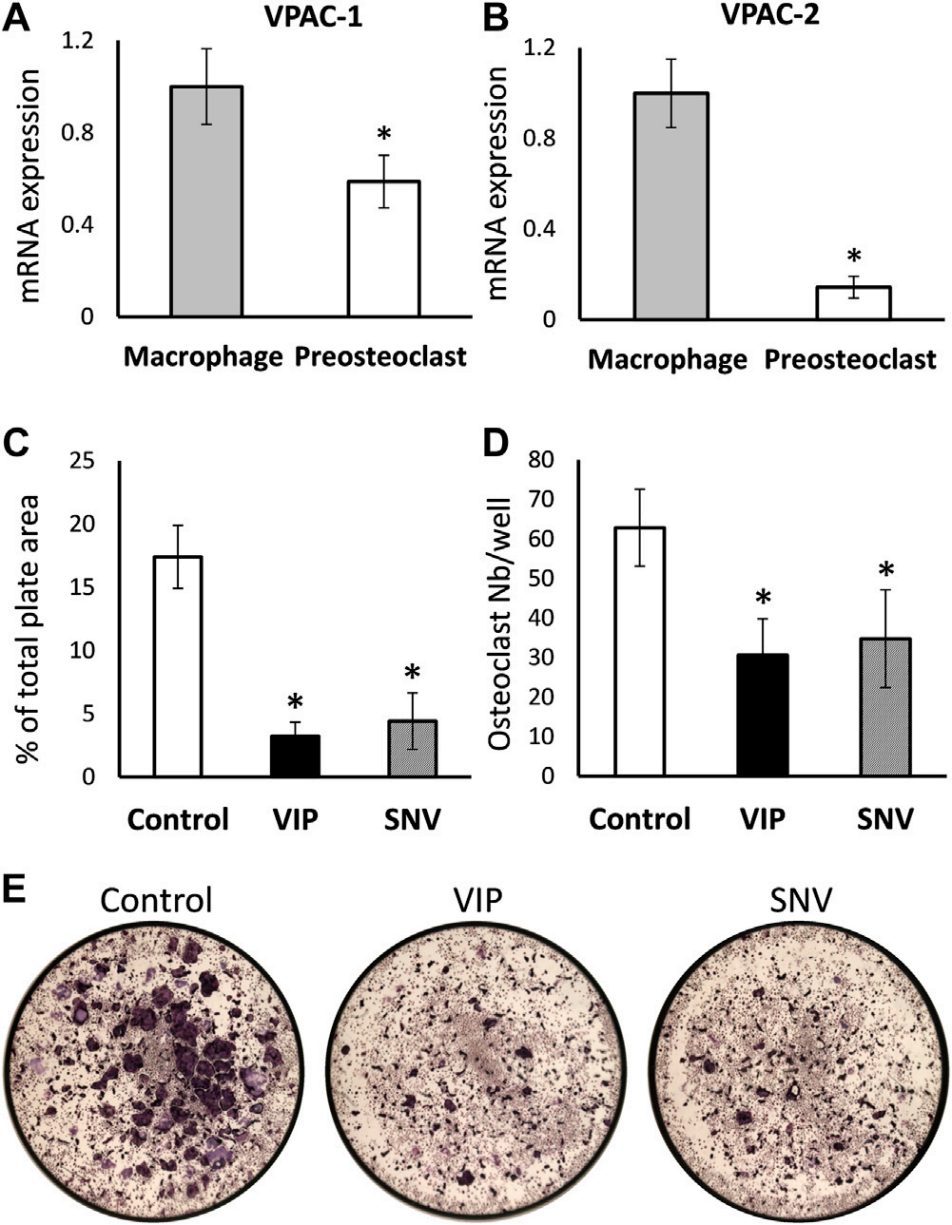
Direct effect of VIP and SNV on osteoclastogenesis. **(A)** VPAC1 and **(B)** VPAC2 expression in M-CSF treated bone marrow-derived macrophages (Day 0) and M-CSF + RANKL treated pre-osteoclasts (Day 2) using RT-qPCR. Data are Mean±SD, normalized to expression levels in macrophages, performed in triplicates in 2 independent experiments with similar results. *, *p*<0.05 versus macrophages. **(C-E)** Osteoclastogenesis in the presence of 10^-6^M VIP, SNV or vehicle only (control) performed in 96-well plates; TRAP-positive multinucleated osteoclasts were evaluated after 5 days of differentiation. **(C)** Osteoclast % surface area and **(D)** number per well, Data are Mean±SD, *n*=5, *, *p*<0.01, ANOVA. **(E)** Representative images taken at a 2× original magnification (entire surface of well = 0.32 cm^2^).

We then examined the direct effect of VIP and SNV on osteoclastogenesis. First, we asked whether the transcripts coding for VPAC-1 and VPAC-2, the classical receptors for VIP and SNV, are expressed in preosteoclasts during the differentiation process. BMDM, which are osteoclasts precursors *in vitro*, were cultured in the presence of M-CSF and RANKL to induce osteoclast differentiation. VPAC-1 and VPAC-2 transcripts were both expressed in BMDM before the addition of RANKL (Day 0) and these levels declined by 58 and 92%, respectively, within 2 days of osteoclastogenesis (Figures 3A,B). We then assessed the effect of VIP and SNV on osteoclast differentiation in the absence of inflammation. In line with previous reports (Mukohyama et al., 2000) (Lundberg et al., 2000), VIP significantly inhibited osteoclastogenesis as indicated by the reduced osteoclast number and total area (Figures 3C–E). SNV had a comparable inhibitory effect on osteoclast differentiation with no difference between the effect of VIP and that of SNV in this assay.

**FIGURE 4:**
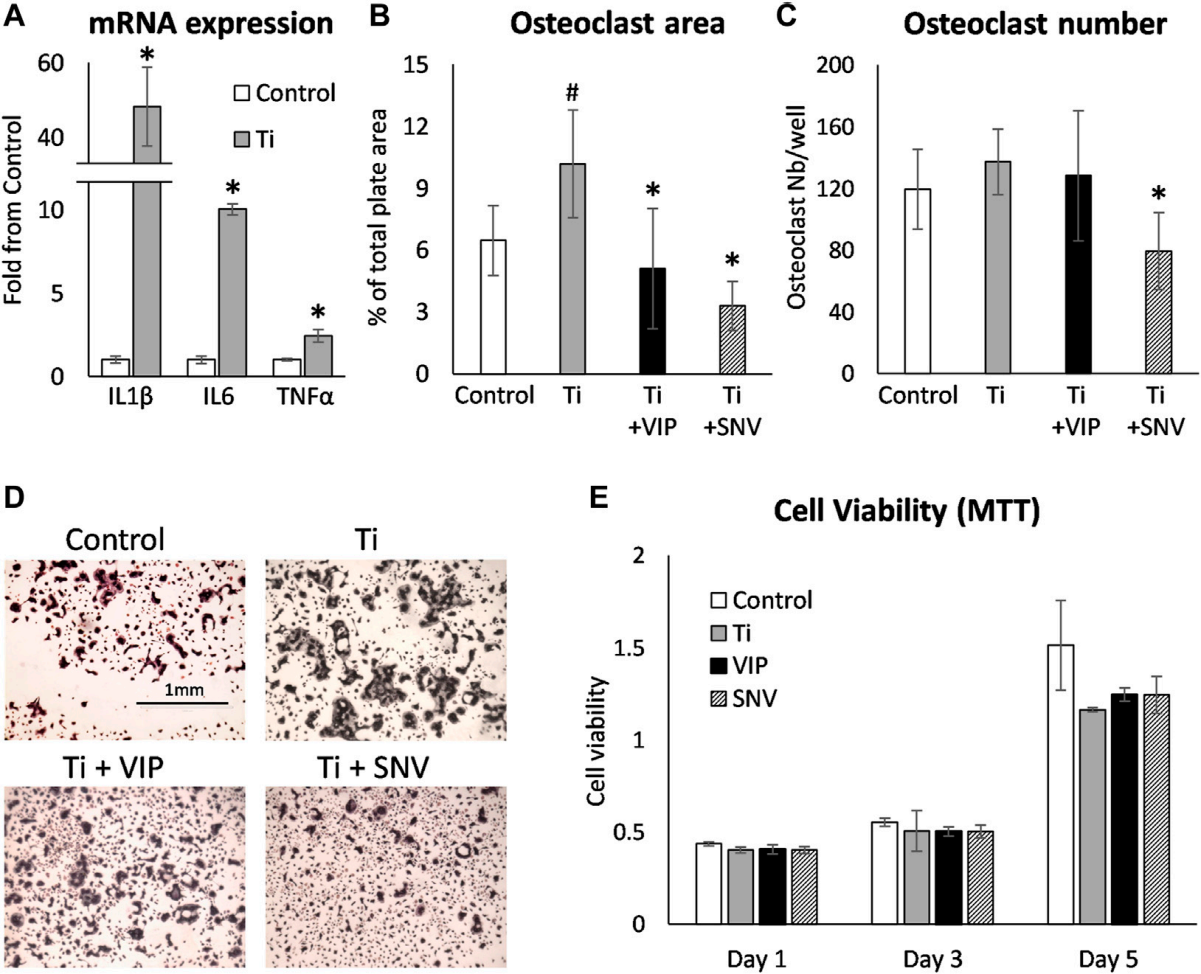
VIP and SNV alleviate the osteoclastogenic effect of TiP-induced inflammation. (A) Primary BMDM were exposed to TiP and RNA was collected after 24 h. mRNA expression are presented as Mean±SD fold change versus Control. * *p*<0.05 vs. Control. **(B-D)** Primary murine preosteoclasts were cultured in 20 ng/ml M-CSF and 50 ng/ml RANKL for 48 h. Conditioned medium from macrophages (Control) and macrophages exposed to Ti particles (Ti, as in A) was then added together with 10^-6^M of VIP or SNV (or saline) for 48 h before TRAP staining. Data are Mean±SD for osteoclast % surface area **(B)** and number per well **(C)**. **(D)** Representative images all acquired at a 4x original magnification. The scale bar for all images is in the upper left panel. *n*=8, #*p*<0.05 vs. Control; *p<0.05 vs. Ti, ANOVA. **(E)** Primary murine macrophages/preosteoclasts were cultured in 20 ng/ml M-CSF and Ti particles, 10^-6^M of VIP or SNV, or saline only. MTT viability assay was performed after 1, 3 and 5 days and presented as optical density values. *N*=5, no statistical differences were found between the treatment groups. Data are Mean±SD in arbitrary OD units.

We next exposed BMDM to TiP and as previously reported by us, we found that IL1β, IL6 and TNFα were significantly elevated, similarly to the response to LPS (Figure 2, 4A). We also collected the supernatant from macrophages exposed to TiP and added it to day-2 osteoclastogenic cultures together with VIP, SNV or no treatment (Figures 4B–D). Notably, the supernatant of TiP-exposed macrophages had a significant effect on osteoclastogenesis, increasing osteoclast area by 57%. In these inflammatory settings, VIP treatment reduced osteoclast area and SNV attenuated both osteoclast area and number, displaying a stronger inhibitory effect on osteoclasts than VIP. This inhibitory effect was not attributable to cytotoxicity as neither VIP nor SNV affected the viability of the cells in the culture over 5 days of treatment (MTT assay, Figure 4E).

### SNV Is Skin Bioavailable

**FIGURE 5:**
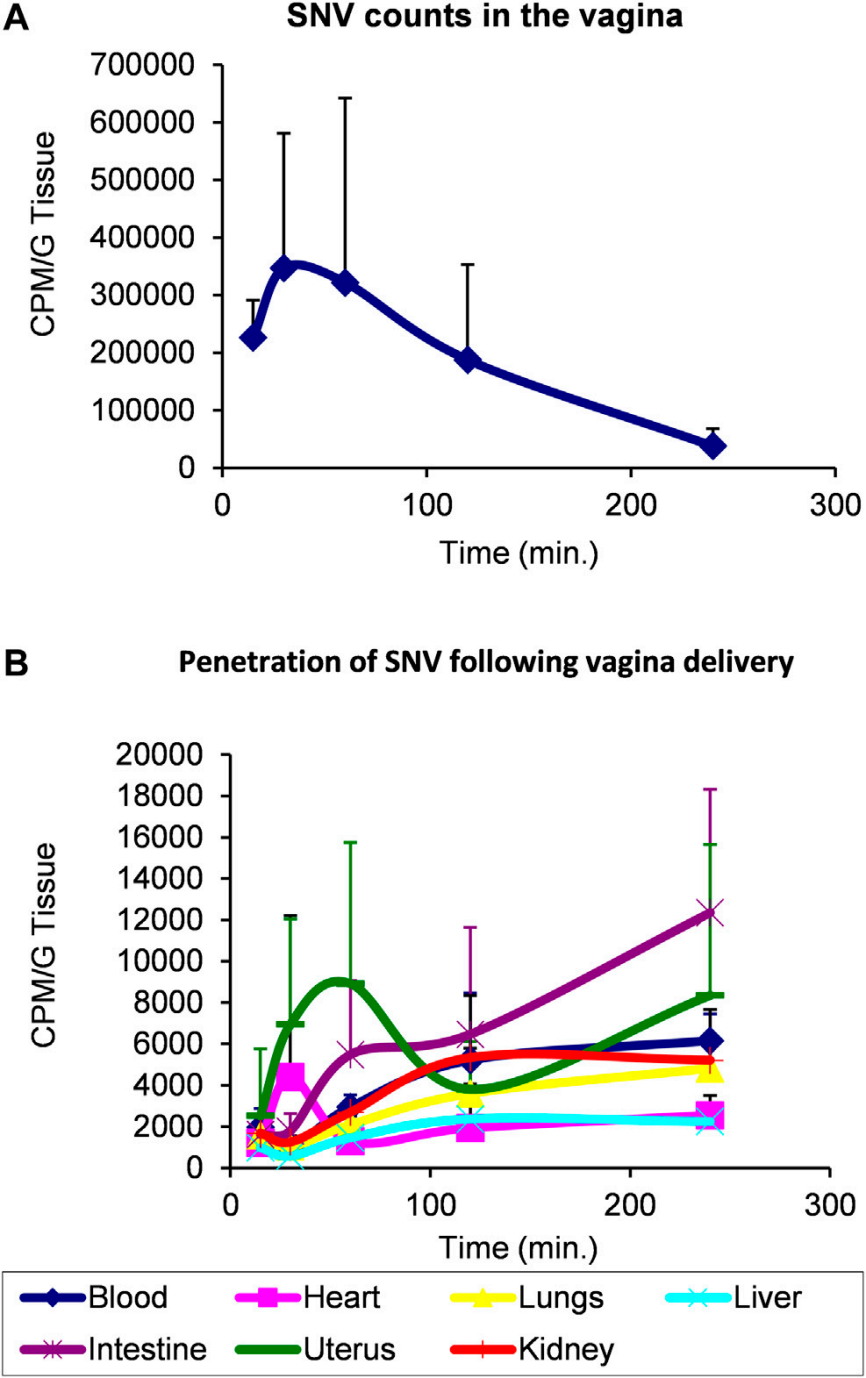
SNV demonstrated a high transepithelial penetrability and stability using a model of vaginal delivery in rats. 7.0 microCi/rat (15 million CPM, 100 Ci/mmole specific activity) of radioactive SNV was delivered in the vagina of rats at T0. Each point on the chart represents at least two rats. Levels of radioactivity were monitored over 250 min to assess the remaining amount of SNV in the vagina **(A)** versus its distribution in the indicated organs **(B)** as a measure of its penetrability through the vaginal epithelium. Data are Mean ± SD.

Based on these findings, and due to former pharmacological experiments indicating that the therapeutic applications of VIP have been hampered by its very short half-life and low penetration through lipidic barriers (skin and epithelium), we elected SNV as a potential therapeutic agent in the treatment of inflammation-induced osteolysis. A prerequisite for such a treatment modality is high penetrability through tissues and epithelium. We therefore conducted a transepithelial penetrability assay in a rat model. In this assay, a bolus of radiolabeled SNV was delivered to the vagina and the local levels of SNV showed a decline over time (Figure 5A). In parallel, the levels of SNV in the blood, heart, lungs, liver, gut, uterus and kidneys steadily increased (Figure 5B), indicating the high penetrability of SNV through the vaginal epithelium, and further tissue stability. The integrity of the radioactive SNV was previously shown (Gozes et al., 1996).

### SNV Is Nontoxic

We also conducted several toxicology studies in rabbits, guinea pigs and rats as detailed in the method section, and all showed a clean toxicology profile (Table 1). The study named 004-1, was the most protracted and detailed toxicology study. We carried it out in rats as a repeated dose toxicity study on target external organs, the penis and the vagina. The structure of the study included a detailed pathological evaluation of all body organs.

No drug-related macroscopic or microscopic changes were detected in any of the dosages used. All changes reported by the pathologist were regarded as spontaneous incidental pathological findings similar to those not infrequently found in rats of the same strain and age used as controls.

### SNV Protects Again TiP-Induced Osteolysis

**FIGURE 6:**
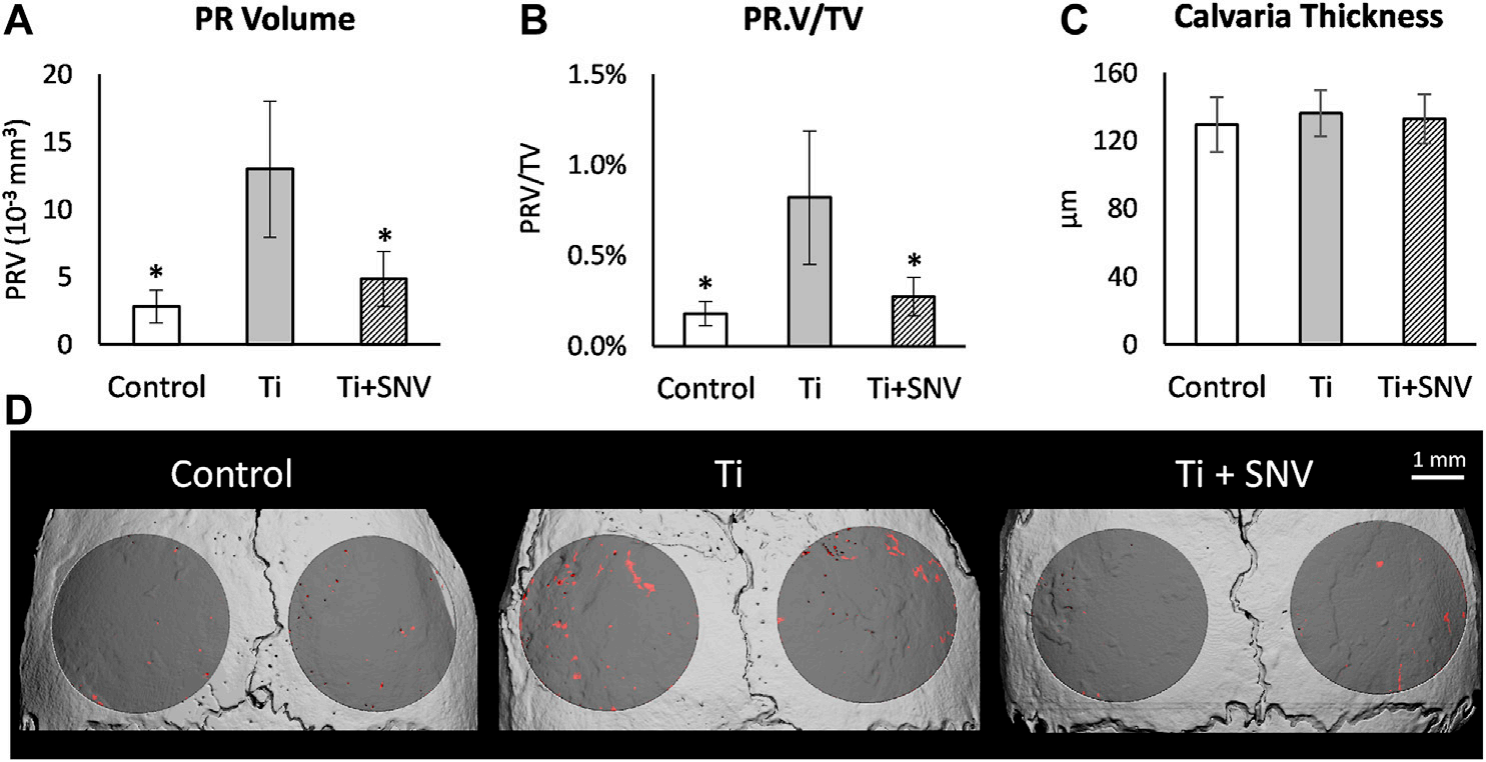
Effect of topically-administered SNV on particle-induced osteolysis *in vivo*. Fibrin membranes including titanium particles, together with SNV (or saline as control) were implanted onto mouse calvaria. Mice were euthanized 5 weeks post-op for μCT analysis. **(A)** Pit resorption volume (PRV, mm^3^), **(B)** PRV/TV (%), **(C)** Calvarial thickness (μm). Data are expressed as mean±SD, *n*=6-9, *p<0.05 vs. Ti, ANOVA. **(D)** Representative μCT images of the calvaria. The region of interest (ROI) is represented in dark gray, and the resorption pits are denoted in red. Bar = 1 mm.

Next, we conducted a TiP-induced osteolysis assay using our mouse calvarial model (Eger et al., 2017; Eger et al., 2018). The topical effect of SNV was tested by incorporating SNV into membranes that were also loaded with Ti particles. We assumed that SNV will penetrate the tissues and suppress the inflammation and osteoclastogenesis on the calvarial surface. In this experiment, we observed a severe osteolysis on the calvarial surface, beneath the TiP-loaded membrane (Figure 6). Topically administered SNV significantly suppressed the TiP-induced osteolysis leading to a 60% significantly lower pit resorption volume and pit resorption volume as percentage of the total tissue volume (Figures 6A,B). There were no significant differences in the calvaria thickness (Figure 6C), supporting the idea that SNV suppressed pit resorption rather than stimulated irregular bone apposition.

## Discussion

VIP and its derivative SNV have been previously studied for immunomodulation properties (Abad et al., 2006; Goursaud et al., 2015). Here we show the effect of each peptide on the cytokine profile of macrophages in response to LPS and in the osteolytic response to Ti particles using *in vitro* and *in vivo* mouse models. Our data indicated that under the currently described experimental conditions, SNV had a stronger anti-inflammatory and anti-osteoclastogenic effect than VIP *in vitro*, in the presence of LPS and TiP, respectively. Furthermore, loading a single dose of SNV into a degradable membrane significantly inhibited bone resorption in a mouse calvarial model of inflammation-induced osteolysis. Lastly, this study also demonstrated the safety profile and high transepithelial penetrability of SNV. The presented data also shows the bioavailability of radioactive SNV (Figure 5), in line with the previously published half-life of SNV (Gozes et al., 1996) and rapid clearance of intravenously administered SNV (10% remaining intact SNV, 30 min after injection (Gozes et al., 1994).

Although SNV is derived from VIP, differences were reported in terms of potential mechanisms (cAMP vs. cGMP, see above), and biological potency in models such as neuroprotection (Gozes et al., 1995; Delgado et al., 1998; Ashur-Fabian et al., 1999; Kim et al., 2000). These and other previous studies showed that VIP downregulates TNFα expression in LPS-treated RAW264.7 cells and activated microglia, and *in vivo* in models of nerve injury (Kim et al., 2000) and endotoxemia (Delgado et al., 1999). In contrast, in our study VIP had no significant effect on TNFα expression in LPS-treated primary BMDM (Figure 2). This apparent contradiction may stem from the origin of the macrophages (BMDM vs. RAW264.7 and rat microglia) or from indirect mechanisms that only take place *in vivo*.

Importantly, previous studies showed that VIP may present a cAMP-dependent pro-inflammatory role (Vu et al., 2014) and induce IL6 expression (Mullol et al., 1997; Martinez et al., 1998; Brenneman et al., 2003) while SNV does not activate cAMP formation (Goursaud et al., 2015). Here also, we found that VIP increased IL6 expression in LPS-activated macrophages, whereas SNV did not (Figure 2), supporting the notion of a distinct mechanism between VIP and SNV. The superior anti-osteoclastogenic effect of SNV over VIP may therefore result from either its inhibitory effect on IL1β with no stimulation of IL6, a separate internal signaling pathway or its longer half-life (Gozes et al., 1996). Another advantage of SNV is that its chemical properties allowed its incorporation in the thrombin-fibrinogen membrane whereas VIP and its solvent perturbed the formation of the membrane (data not shown). Notably, we cannot rule out that more stable delivery methods of VIP would improve its bioactivity as reported by others (Wang et al., 2016).

Previous studies showed controversial data regarding the effect of VIP on osteoclastogenesis. VIP was shown to stimulate bone resorption in an *ex vivo* organ culture (Hohmann et al., 1983) but had no effect on basal osteoclastogenesis and even inhibited osteoclast formation in mouse bone marrow cultures (Mukohyama et al., 2000). These opposing findings may be rooted in the fact that VIP has a pro-osteoclastogenic effect mediated by its actions on osteoblasts but also an anti-osteoclastogenic effect via its direct action on osteoclasts (Lundberg et al., 2000). Our data further support the conclusion of such a direct inhibitory effect of VIP on pre-osteoclasts. In our inflammatory models *in vitro*, the observed impact of VIP on osteoclast differentiation is likely a direct anti-osteoclastogenic effect that slightly supersedes the pro-osteoclastogenic increase in IL6. The therapeutic potential of VIP has been tested in a rat model of periodontitis and displayed a partial effect on the inflammatory status and osteoclastogenic signals but no significant positive outcome on bone loss (Gürkan et al., 2009). In contrast, we show here that SNV significantly prevented the bone loss induced by the presence of TiP. Our findings suggest that while SNV and VIP have similar effects on osteoclast differentiation (Figure 3), SNV exerts a more potent anti-osteoclastogenic effect in the context of inflammation (Figure 4). One of our study limitations is that we did not directly demonstrate the effect of SNV *in vivo* on the inflammatory response and in a model of bacteria-induced osteolysis. It should be emphasized that a corroborative independent study demonstrated the significant anti-inflammatory effects of SNV *in vivo* in an amyotrophic lateral sclerosis (ALS) model (Goursaud et al., 2015). In our study, the strong suppression of osteoclast differentiation (*in vitro*) and bone resorption (*in vivo*) by SNV might result from the combined effect on the inflammation (decrease in IL1β, Figure 2 and (Eger et al., 2018)) as well as a direct effect on osteoclasts (Figure 3). Notably, our osteoclastogenic assay performed in the presence of conditioned medium from TiP-exposed macrophages (Figure 4), contained a host of pro-inflammatory and pro-osteoclastogenic signals, and SNV blocked or supplanted these signals, pointing to a strong inhibitory effect on inflammation-induced osteoclast differentiation.

Both SNV and VIP bind at VPAC-1 and VPAC-2 (Gourlet et al., 1998) and macrophages from humans and mice express both receptors (Calvo et al., 1994; Herrera et al., 2009; Burian et al., 2010; Hauk et al., 2014). Others showed that VPAC-2 expression increases in LPS-exposed macrophages (Herrera et al., 2009) and we showed that VPAC-2 expression declines during osteoclast differentiation (Figure 3), in line with previous studies showing that osteoclasts mainly express VPAC-1 (Ransjö et al., 2000). On the other hand, our previous work (Delgado et al., 1998) in RAW264.7 murine macrophages showed that VIP inhibited LPS-driven TNFα production via VPAC-1. Notably, SNV exhibits a differential affinity to human and rodent VPAC-1 and VPAC-2 (Gourlet et al., 1998), suggesting that the ratio of expression of the two receptors in rodent and human cells will dictate the biological outcome and mechanism of action of VIP and SNV in inflammation and osteoclastogenesis in humans. A very recent study showed that VIP further modulates human macrophage phenotype via formyl peptide receptor-like 1 (FPRL1) and activation of RhoA-GTPase and PLC pathways (Harhous et al., 2021), while both VIP and SNV act through cGMP to increase neuroprotection (Ashur-Fabian et al., 1999). These findings pave the path for future mechanistic evaluations.

As discussed above, inflammation-induced osteolysis is the common pathological outcome of various conditions including periodontitis, oral peri-implantitis and orthopedic implant loosening. In this study we showed using distinct *in vitro* assays that SNV blocked the two common denominators leading to osteolysis, i.e. inflammation and osteoclastogenesis, notwithstanding the cause of the inflammation, i.e. bacterial (LPS) or aseptic (TiP), clearly demonstrating a greater therapeutic potential than VIP. In both oral periimplantitis and orthopedic periprosthetic osteolysis, macrophages contribute to the inflammatory response and secretion of pro-osteoclastogenic signals (Revell, 2008; Fretwurst et al., 2020). In future studies, it would be interesting to test the therapeutic effect of SNV in gingival fibroblasts and periodontal ligament cells that also fuel the inflammation and bone loss around oral implants. Obviously, our observations obtained using *in vitro* and *in vivo* models also warrant further investigation using various bacterial strains known for their deleterious impact on bone, at different anatomical sites (e.g. jaw, hip), as well as in human cells and validation in placebo controlled double-blind randomized clinical trials.

This study attributes for the first time a therapeutic potential to SNV in the treatment of inflammation-induced osteolysis. Its high penetrability and low systemic toxicity portray SNV as a valid candidate for topical administration. Our previous studies showed a close match between the inflammatory response induced by Ti particles and that induced by LPS, suggesting that SNV presents a promising therapeutic approach in the management of peri-prosthetic and periodontal bone loss induced by wear particles and bacterial infection.

## Data Availability

The original contributions presented in the study are included in the article/Supplementary Material, further inquiries can be directed to the corresponding authors.
